# Highly Efficient Biotransformation of Polydatin to Resveratrol by Snailase Hydrolysis Using Response Surface Methodology Optimization

**DOI:** 10.3390/molecules18089717

**Published:** 2013-08-13

**Authors:** Zi Wang, Li-Chun Zhao, Wei Li, Lian-Xue Zhang, Jing Zhang, Jian Liang

**Affiliations:** 1 College of Chinese Medicinal Materials, Jilin Agricultural University, Changchun 130118, China; 2 College of Vocation and technology, Changchun University of Science and Technology, Changchun 130600, China; 3 The Affiliated Ruikang Hospital, Guangxi University of Chinese Medicine, Nanning 530011, China

**Keywords:** snailase hydrolysis, polydatin, resveratrol, response surface methodology

## Abstract

Resveratrol (RV), a dietary antioxidant polyphenolic compound found in grapes and red wine, exerts a wide variety of pharmacological activities. However, lower content in plants compared with polydatin (PD, the glucoside of RV) limits its application in the food and pharmaceutical industries. In this paper, we carried out efficient biotransformation of PD to RV with 100% conversion yield by snailase hydrolysis. Moreover, response surface methodology (RSM) was used to optimize the effects of the reaction temperature, enzyme load, and reaction time on the conversion process. Validation of the RSM model was verified by the good agreement between the experimental and the predicted RV yield values. The optimum preparation conditions were as follows: temperature of 62.0 °C, enzyme load of 6.6%, and reaction time of 96 min. The proposed method may be highly applicable for the enzymatic preparation of RV for medicinal purposes.

## 1. Introduction

**Figure 1:**
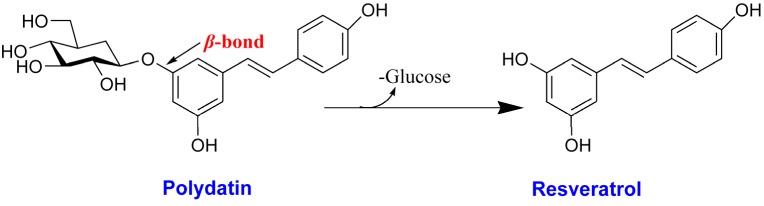
The structures of polydatin and resveratrol, and their biotransformation reaction.

Resveratrol (3,5,4′-trihydroxy-*trans*-stilbene, RV) is a polyphenolic compound found in a variety of plant species, including *Polygonum cuspidatum*, grapes, berries, peanuts and other foodstuffs [1]. However, resveratrol mostly exists in plants as its glycoside form, polydatin [2]. After oral administration in humans, polydatin is metabolized in the small intestine, and converted RV is the final active form absorbed across intestinal Caco-2 cells [3,4,5] (Figure 1). Recently, RV has received increasing attention because of its several reported beneficial effects including cardiovascular diseases treatment [6,7], anti-cancer [8,9], antioxidant activity [10,11], and hypoglycemic activities [12,13].

Due to its prophylactic or therapeutic value to human subjects, it is essential to employ an effective method to obtain active RV. Polydatin, the glycoside form of RV, is present at a much higher content (2%) in dried root of *P*. *cuspidatum* than in wine and other sources [14]. Therefore, this herb is a better material source for production of RV. In a previous investigation, large amounts of crude polydatin were isolated and purified from *P*. *cuspidatum.* In general, resveratrol can be obtained by chemical synthesis [15], solvent extraction [16,17], and microorganism transformation [18,19,20,21,22]. Prior to the present investigation, Doehlert design was used to optimize the liquid–liquid extraction of resveratrol from wine by an organic solvent [23]. Although the production of RV through the above methods is reasonable and feasible, these processes generate large amounts of waste organic solvents and involve lengthy operation techniques. The higher selectivity and efficiency of enzymatic preparations can overcome the limitations of chemical synthesis and provide an important green tool to obtain RV within a shorter time, unlike microbial transformation [24,25]. Enzymatic preparation has been suggested to be the most efficient transformation technique for target compounds production.

Recently, snailase (a complex of cellulase, hemicellulase, pectinase and β-glucuronidase), extracted from the digestive tract of snails, has received increasing attention due to strong hydrolysis ability [26,27]. In previous papers, we have employed snailase to produce ginsenoside and platycodins with great success [28,29]. In this work, we report a highly efficient biotransformation of polydatin to RV using snailase hydrolysis. Moreover, response surface methodology (RSM) was employed as an effective statistical technique to evaluate the hydrolysis parameters and their interactions by establishing a mathematical model. The effects of reaction temperature, enzyme load, and reaction time on the snailase hydrolysis efficiency and their interactions were systemically analyzed for the first time with the RSM method.

## 2. Results and Discussion

### 2.1. Model Fitting

After the preliminary ranges of the preparation variables were determined by one-factor-at-a-time experiments, the three independent variables: the reaction temperature (*X_1_*, 40–70 °C), enzyme load (*X_2_*, 2%–10%) and reaction time (*X_3_*, 30–180 min), were fixed to optimize the yields of RV. The preliminary test gave the presence of reaction equilibrium at around 180 min. Taking into account this reaction equilibrium, we also set enzyme load at a range of 2 to 10% and temperature at the range of 40 to 70 °C, respectively. The whole design consisted of 17 experimental points as listed in Table 1, and five replicates (run 13–17) at the center of the design were used for estimating the experimental error sum of squares. The triplicates were performed at all design points in randomized order. Box-Behnken design (BBD), as one of the RSM designs, is applied in the present study.

**Table 1:** Box-Behnken experimental design with the independent variables.

Run	Coded variables levels			*Y*	
				RV (mg/mL)	
	*X*_1_, Reaction temperature (°C)	*X*_2_, Enzyme load (%)	*X*_3_, Reaction time (min)	Actual	Predicted
1	40.00	2.00	105.00	9.50	10.10
2	70.00	2.00	105.00	8.82	8.81
3	40.00	10.00	105.00	8.64	8.65
4	70.00	10.00	105.00	12.06	11.47
5	40.00	6.00	30.00	11.70	11.46
6	70.00	6.00	30.00	12.13	12.50
7	40.00	6.00	180.00	11.88	11.52
8	70.00	6.00	180.00	11.77	12.01
9	55.00	2.00	30.00	10.08	9.72
10	55.00	10.00	30.00	9.61	9.84
11	55.00	2.00	180.00	9.25	9.02
12	55.00	10.00	180.00	9.76	10.11
13	55.00	6.00	105.00	13.39	13.06
14	55.00	6.00	105.00	13.36	13.06
15	55.00	6.00	105.00	12.78	13.06
16	55.00	6.00	105.00	12.85	13.06
17	55.00	6.00	105.00	12.92	13.06

As Table 2 shows, the analysis of variance (ANOVA) of conversion yield of RV indicated that experimental data had a determination coefficient (*R^2^*) of 0.9604 with the calculated model with no significant lack of fit at *p* > 0.0001 (*p* = 0.0063). That means that the calculated model was able to explain 96.04% of the results [30]. The results indicated that the model used to fit response variables was significant (*p* < 0.0001) and adequate to represent the relationship between the response and the independent variables. *F*-test suggested that model had a very high model *F*-value (*F* = 18.89), indicating this model was highly significant. *R*^2^_adj_ value (adjusted determination coefficient) is the correlation measure for testing the goodness-of-fit of the regression equation [31]. The *R*^2^_adj_ value of this model is 0.9096, which indicated only 9.04% of the total variations were not explained by model. Meanwhile, a relatively lower value of coefficient of variation (CV = 4.50) showed a better precision and reliability of the experiments carried out.

**Table 2:** Analysis of variance for the fitted quadratic polynomial model.

Source	SS	DF	MS	*F*-value	Prob > F	
Model	43.27	9.00	4.81	18.89	<0.0001	significant
Residual	1.78	7.00	0.25			
Lack of fit	1.44	3.00	0.48	5.69	0.0063	insignificant
Pure error	0.34	4.00	0.08			

SS, sum of squares; DF, degree of freedom; MS, mean square.

It can be seen in Table 3 that conversion yield of RV was affected most significantly by reaction temperature (*X*_1_), followed by enzyme load (*X*_2_) and reaction time (*X_3_*). It was also observed that the quadratic parameter of 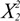 was the most significant at the level of *p* < 0.0001, the others (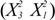) were significant at the level of *P*<0.01. As for interaction quadratic parameters, the interaction effect of *X*_1_*X*_2_ was significant and that of *X*_1_*X*_3_ and* X*_2_*X*_3_ was insignificant (*p* > 0.01). Predicted response *Y* for the yield of RV could be expressed by the following second-order polynomial equation in term of coded values:

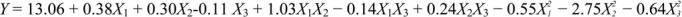
where *Y* is the yield of RV (mg/mL), and *X*_1_, *X*_2_ and *X*_3_ are the coded variables for reaction temperature, enzyme load and reaction time, respectively.

**Table 3:** Estimated regression model of relationship between response variables (*Y*) and independent variables (*X*_1_, *X*_2_, *X*_3_).

Variables	DF	SS	MS	*F*-values	*p*-value
*X*_1_	1	1.17	1.17	4.60	0.0692
*X*_2_	1	0.73	0.73	2.86	0.1348
*X*_3_	1	0.09	0.09	0.37	0.5639
*X*_1_*X*_2_	1	4.21	4.21	16.55	0.0048
*X*_1_*X*_3_	1	0.07	0.07	0.29	0.6091
*X*_2_*X*_3_	1	0.24	0.24	0.93	0.3674
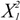	1	1.29	1.29	5.09	0.0588
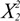	1	31.85	31.85	125.16	<0.0001
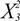	1	1.70	1.70	6.68	0.0362

### 2.2. Analysis of Response Surface

**Figure 2:**
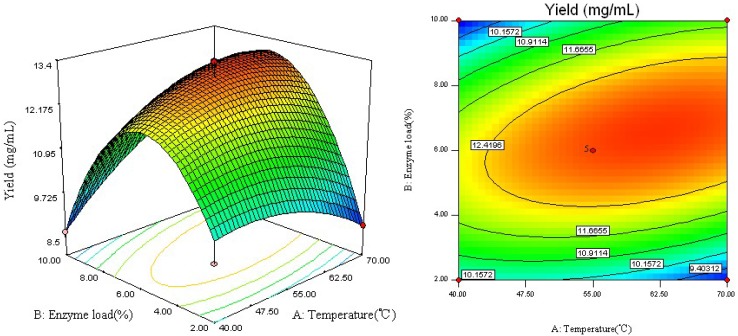
Response surface plot and contour plot of reaction temperature and enzyme load.

**Figure 3:**
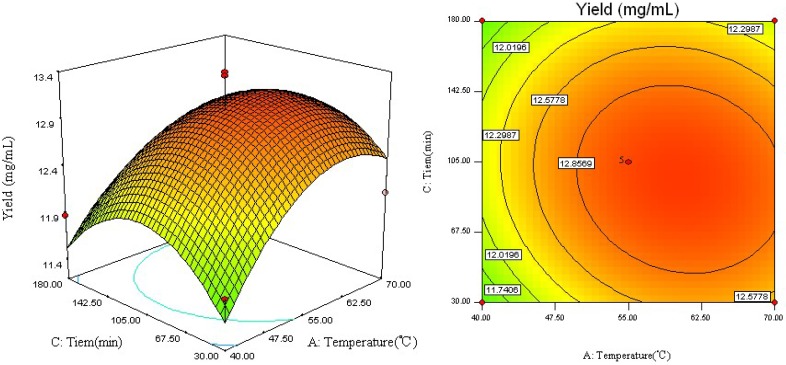
Response surface plot and contour plot of reaction temperature and reaction time.

The regression equation was graphically represented by a 3D response surface and 2D contour plots. From three dimensional response surface curves and contour plots shown in Figure 2 and Figure 3, the effects of the independent variables and their mutual interaction on the yield of RV can be seen. Figure 2 shows the effect of the interaction between reaction temperature (*X*_1_) and enzyme load (*X*_2_) on the RV yield. An increase in reaction temperature from 40 to 55 °C with an enzyme load increase from 2 to 6% enhanced the conversion yield of RV, while with any increase of the reaction temperature over 55 °C, there was a gradual decline in the response and enzyme loads over 6.5% did not show any obvious effect on the yield of RV.

Figure 3 depicts the effect of reaction temperature (*X*_1_) and reaction time (*X*_3_) on the yield of RV. As shown in Figure 3, it may be observed that with an increase of reaction temperature from 40 to 65 °C and reaction time from 30 to 100 min, the yield of RV was increased gradually. However, there was also a decrease in RV yield over a reaction temperature of 65 °C and with reaction times of more than 100 min.

**Figure 4:**
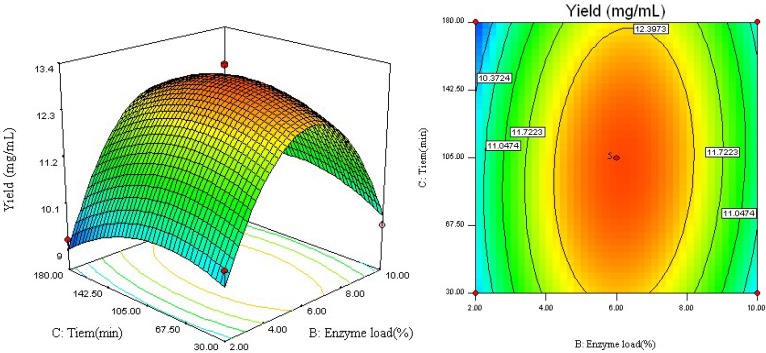
Response surface plot and contour plot of enzyme load and reaction time.

Figure 4 displays the effect of the interaction of enzyme load (*X*_2_) and reaction time (*X*_3_) on the yield of RV. It shows that the highest conversion yield could be achieved when using about 6.5% of enzyme load and 105 min of reaction time. However, the conversion yield did not increase with enzyme loads over 6.5%. Moreover, 105 min of reaction time is enough for enzymatic preparation to convert all polydatin to RV.

### 2.3. Optimal Conditions and Model Verification

The optimal conditions obtained using the models were as follows: reaction temperature of 62.35 °C, enzyme load of 6.57% and reaction time of 96.76 min, respectively. The software-predicted yield of RV was 13.18 mg/mL. In order to compare the predicted result with the practical value, three parallel experiments were carried out using the optimal conditions, as shown in Table 4. An average value of 12.88 ± 0.27 mg/mL was observed from real experiments, which is in close agreement with the value predicted by the model equation. This indicated that the optimization presented in the present study is reliable.

**Table 4:** Optimum conditions and the predicted and experimental value of response at the optimum conditions.

	Reaction temperature (°C)	Enzyme load (%)	Reaction time (min)	Yield of RV (mg/mL)
Optimum conditions	62.35	6.57	96.76	13.18 (predicted)
Modified conditions	62.0	6.6	96.0	12.88 ± 0.27 (actual)

## 3. Experimental

### 3.1. Materials and Chemicals

Polydatin was prepared in our lab and its structure was elucidated on the basis of spectroscopic methods, including UV, IR, MS, and ^13^C-NMR. Snailase was purchased from Beijing Biodee Biotechnology Co., Ltd. (Beijing, China). HPLC-grade acetonitrile and methanol were purchased from Fisher Chemicals (Waltham, MA, USA). Other chemicals were all of analytical grade and obtained from Beijing Chemical Factory (Beijing, China).

### 3.2. Isolation and Purification of Polydatin

Dried roots of *P. cuspidatum* (500 g) were refluxed three times with 15 L of 80% ethanol. Extracts were concentrated and suspended in water. The aqueous layer was subjected to macroporous resin AB-8 column chromatography eluting sequentially with water and 30% ethanol. The 30% ethanol elutate was repeatedly chromatographed on a reverse-phase column eluting with aqueous methanol to afford crude polydatin (8.5 g).

### 3.3. Enzymatic Preparation of RV from Polydatin

Snailase was incubated with polydatin in a pH 4.5 sodium acetate buffer with agitation at different temperatures (varying from 40 to 70 °C) and different enzyme loads (varying from 2 to 10%) for a certain time (varying from 30 to 180 min). The mixtures were subsequently placed in a water bath at 90 °C to terminate the enzymatic reaction. The reaction mixtures were individually evaporated, dissolved in methanol, and filtered through a 0.45 μm nylon filter membrane prior to injection into the HPLC system. The chromatographic peaks of polydatin and RV were confirmed by comparing their retention times with those of the reference standards. Quantification was carried out by the integration of the peak using external standard method.

### 3.4. HPLC analysis of the Bioconversion Process

**Figure 5:**
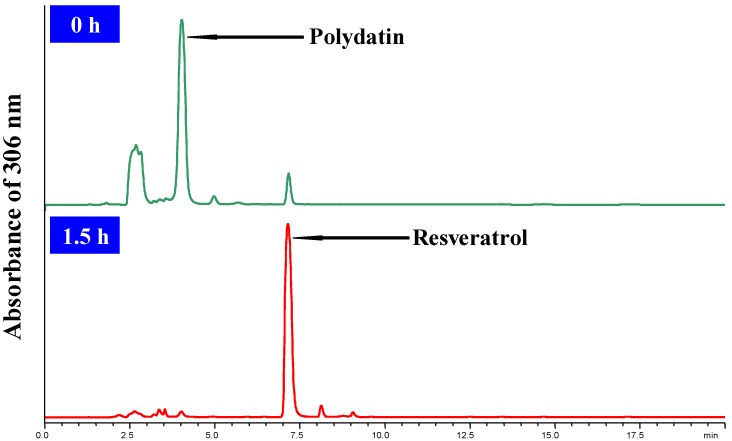
HPLC analysis of the bioconversion of polydatin to resveratrol.

The HPLC analysis was performed with a HPLC instrument (Agilent 1100, Santa Clara, CA, USA) equipped with a quaternary solvent delivery system, a column oven and UV detector. A HPLC method was developed using a reversed-phase C18 column (Hypersil ODS2, 250 mm × 4.6 mm I.D., 5 μm). The column temperature was set at room temperature and detection wavelength was set at 300 nm. The mobile phase was consisted of 25% acetonitrile with flow rate of 1.0 mL/min. The 10 μL of sample solution was directly injected into the chromatographic column manually. HPLC chromatograms are shown in Figure 5.

### 3.5. Experimental Design

In the present investigation, we employed the software Design Expert (Trial Version 7.1.6, Stat-Ease Inc., Minneapolis, MN, USA) for experimental design, data analysis and model building. A Box-Behnken design (BBD) with three variables was used to determine the response pattern and then to establish a model. Experimental data were fitted to a quadratic polynomial model and regression coefficient obtained. The non-linear computer-generated quadratic model used in the response surface was as follows:
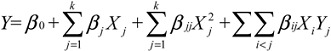
where *Y* is the estimated response, *β_0_*, *β_j_*, *β_jj_* and *β_ij_* are the regression coefficients for intercept, linearity, square and interaction, respectively, while *X_i_*, *X_j_* are the independent coded variables.

### 3.6. Data Analysis

Data were expressed as standard errors of the means (SEM) of three replicated determinations. The response obtained from each set of experimental design (Table 1) was subjected to multiple non-linear regressions using the Design Expert software. The quality of the fit of the polynomial model equation expressed by the coefficient were checked by *F*-test and *P*-value.

## 4. Conclusions

In the present study, polydatin was converted into resveratrol by snailase hydrolysis for the first time. The conditions for enzymatic preparation of resveratrol were optimized using response surface methodology. The estimated models were able to indicate preparation conditions that allowed superior conversion yield. The highest yields predicted for RV could be attained under optimal conditions including 62 °C of reaction temperature, 6.6% of enzyme load and 96 min of reaction time.
